# Cross-shaped bridging stenting for malignant hilar biliary obstruction with surgically altered anatomy

**DOI:** 10.1055/a-2701-5070

**Published:** 2025-09-30

**Authors:** Yuichi Suzuki, Haruo Miwa, Kazuki Endo, Ritsuko Oishi, Hiromi Tsuchiya, Manabu Morimoto, Shin Maeda

**Affiliations:** 1 26437 Gastroenterological Center, Yokohama City University Medical Center, Yokohama, Japan; 2 Department of Gastroenterology, Yokohama City University Graduate School of Medicine, Yokohama, Japan

Bridging stenting via endosonographically created route (ESCR) is an alternative to endoscopic transpapillary drainage for malignant hilar biliary obstruction (MHBO) 1 2 3 , with recent reports indicating superior stent patency compared to transpapillary multi-stenting 4 . We report a case of MHBO with surgically altered anatomy in whom bridging stenting across multiple metallic stents was successfully performed ( Video 1 ).

Bridging stenting from the ESCR to the B6 across multiple metallic stents offers a novel therapeutic option for complex cases of MHBO.Video 1

A 60-year-old man developed a Bismuth type IIIa stricture due to recurrent ampullary carcinoma after pancreaticoduodenectomy ( Fig. 1 ). Endoscopic ultrasound-guided hepaticogastrostomy was performed to the B2, and uncovered self-expandable metallic stents (UCSEMSs) were placed into the anterior and posterior bile ducts using the partial stent-in-stent method by balloon enteroscopy-assisted endoscopic retrograde cholangiopancreatography. Furthermore, percutaneous transhepatic biliary drainage (PTBD) was required to manage an isolated B6 ( Fig. 2 ). Subsequently, bridging stenting from the ESCR to the isolated B6 was attempted to internalize the PTBD ( Fig. 3 ).

**Fig. 1:**
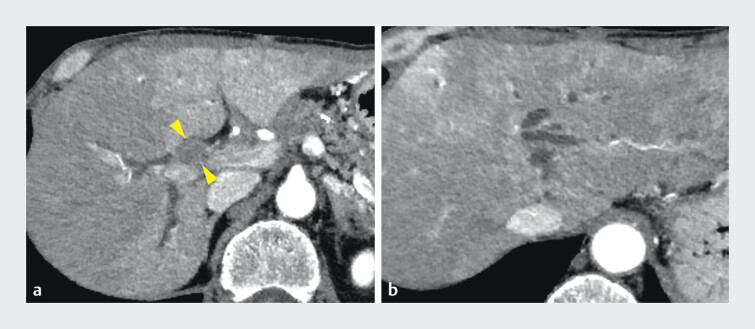
Contrast-enhanced computed tomography (CT) shows a hypovascular tumor at the hepatic hilum (arrowheads) and dilated bilateral intrahepatic bile ducts.

**Fig. 2:**
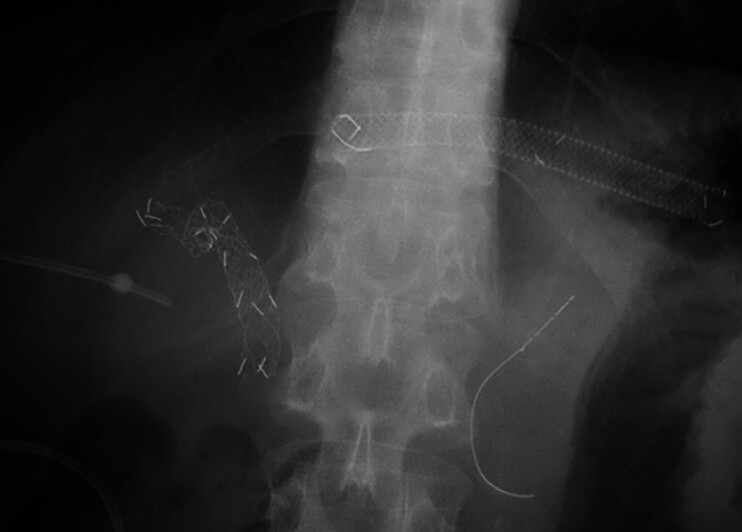
Fluoroscopic image before bridging stenting shows the partially covered self-expandable metallic stent (SEMS) in the endosonographically created route (ESCR) at the B2, two uncovered SEMSs (UCSEMSs) in the anterior and posterior bile ducts, and the percutaneous transhepatic biliary drainage (PTBD) tube in the isolated B6 bile duct.

**Fig. 3:**
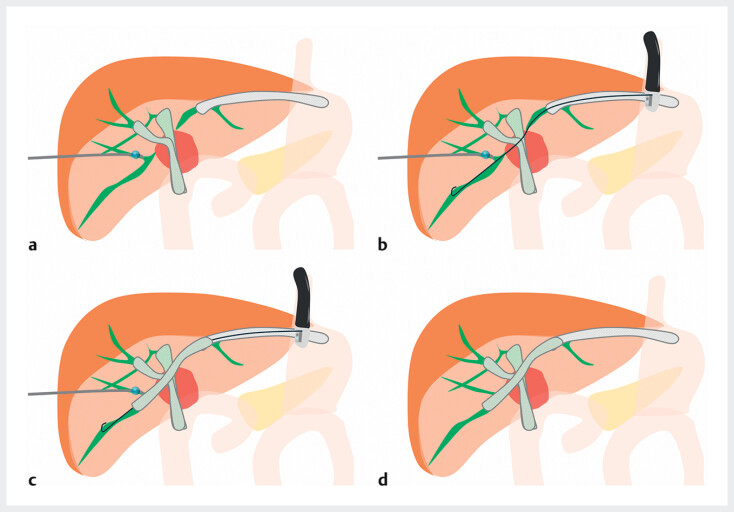
Schemas of bridging stenting from the ESCR to the isolated B6 across multiple metallic stents. **a** The biliary stents and the PTBD tube before the procedure. **b** Guidewire insertion from the ESCR to the isolated B6. **c** An additional metallic stent placement from the ESCR to the B6. **d** Complete internal drainage of the entire liver.

A guidewire was initially advanced into the ESCR; however, seeking the isolated B6 through the mesh of previously placed UCSEMSs was challenging. Therefore, an additional guidewire was advanced via the PTBD route as a landmark, enabling successful insertion of the guidewire from the ESCR to the B6. Although an additional UCSEMS (8 mm × 60 mm; YABUSAME Neo; Kaneka Corporation, Osaka, Japan) was inserted, it failed to pass through the mesh of the UCSEMSs. Therefore, a guide sheath (Endosheather; Piolax, Kanagawa, Japan) was used to facilitate stent insertion, and the UCSEMS was successfully placed from the B6 to the left hepatic duct ( Fig. 4 ). As a result, complete internal drainage of the entire liver was achieved, allowing removal of the PTBD tube and resumption of chemotherapy.

**Fig. 4:**
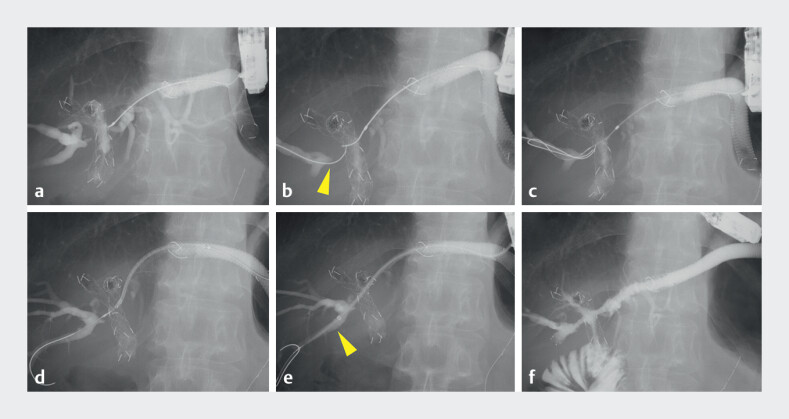
Fluoroscopic images of bridging stenting from the ESCR to the B6 across multiple metallic stents. **a** Seeking the isolated B6 from the ESCR through the mesh of the previously placed uncovered self-expandable metallic stents (UCSEMSs) was challenging. **b** An additional guidewire (arrowhead) was advanced via the PTBD route as a landmark. **c** The guidewire was successfully advanced from the ESCR to the B6. **d** An additional UCSEMS was inserted from the ESCR; however, it failed to pass through the mesh of the UCSEMSs. **e** A guide sheath (arrowhead) was advanced into the B6 through the mesh of the UCSEMSs. **f** The additional UCSEMS was successfully placed through the guide sheath from the B6 to the left hepatic duct. Contrast injection from the ESCR demonstrated a cross-shaped bridging stenting.

To the best of our knowledge, this is the first report of bridging stenting from an ESCR across multiple UCSEMSs, offering a novel therapeutic option for complex cases of MHBO.

Endoscopy_UCTN_Code_TTT_1AS_2AH

## Notes

Endoscopy E-Videos https://eref.thieme.de/e-videos
*E-Videos* is an open access online section of the journal *Endoscopy* , reporting on interesting cases and new techniques in gastroenterological endoscopy. All papers include a high-quality video and are published with a Creative Commons CC-BY license. Endoscopy E-Videos qualify for HINARI discounts and waivers and eligibility is automatically checked during the submission process. We grant 100% waivers to articles whose corresponding authors are based in Group A countries and 50% waivers to those who are based in Group B countries as classified by Research4Life (see: https://www.research4life.org/access/eligibility/ ). This section has its own submission website at https://mc.manuscriptcentral.com/e-videos .
